# Mind Control: How Parasites Manipulate Cognitive Functions in Their Insect Hosts

**DOI:** 10.3389/fpsyg.2018.00572

**Published:** 2018-05-01

**Authors:** Frederic Libersat, Maayan Kaiser, Stav Emanuel

**Affiliations:** Department of Life Sciences and Zlotowski Center for Neurosciences, Ben-Gurion University of the Negev, Beer-Sheva, Israel

**Keywords:** cognition, behavioral manipulation, insects, parasitoids, parasites, hosts, brain

## Abstract

Neuro-parasitology is an emerging branch of science that deals with parasites that can control the nervous system of the host. It offers the possibility of discovering how one species (the parasite) modifies a particular neural network, and thus particular behaviors, of another species (the host). Such parasite–host interactions, developed over millions of years of evolution, provide unique tools by which one can determine how neuromodulation up-or-down regulates specific behaviors. In some of the most fascinating manipulations, the parasite taps into the host brain neuronal circuities to manipulate hosts cognitive functions. To name just a few examples, some worms induce crickets and other terrestrial insects to commit suicide in water, enabling the exit of the parasite into an aquatic environment favorable to its reproduction. In another example of behavioral manipulation, ants that consumed the secretions of a caterpillar containing dopamine are less likely to move away from the caterpillar and more likely to be aggressive. This benefits the caterpillar for without its ant bodyguards, it is more likely to be predated upon or attacked by parasitic insects that would lay eggs inside its body. Another example is the parasitic wasp, which induces a guarding behavior in its ladybug host in collaboration with a viral mutualist. To exert long-term behavioral manipulation of the host, parasite must secrete compounds that act through secondary messengers and/or directly on genes often modifying gene expression to produce long-lasting effects.

## Introduction

The ability of parasites to alter the behavior of their hosts has recently generated an unusual interest in both scientists and non-scientists. One reason is that parasites alter the behavior of their host in such a way as to suggest a hijacking of their ability to make decisions. However, how parasites manipulate their hosts is not an esoteric topic, fascinating with its evocation of gruesome zombie movies involving body snatchers. It is rather the understanding of these processes provide fundamental insights into the neurobiology of behavior. Although our understanding of the neural mechanisms of parasitic manipulation is still lacking, there have been some major advances over the past few years. Since most animals are insects, it is not surprising that many case studies of animals that are manipulated by parasites are insects. The diversity of parasites that can manipulate insect behavior ranges from viruses to worms and also includes other insects that have evolved to become parasites (Hughes and Libersat, 2018). In this short review, we will focus on mind control or the manipulation of cognitive functions in Parasite–Insect associations. We will consider cognition here in a broad sense as the ability of insects to behave not just like reflex machines or automatons (Webb, 2012), but that insects are capable of informed choice-making and goal-directed behavior in a dynamic environment. Recent accumulating evidence demonstrates that insects are more than just automatons and capable of expressing endogenously-created patterns of spontaneous behavior (Perry et al., 2017). For instance, when a single odor is presented to fruit flies in a T-maze at two different concentrations that are easy to tell apart, they make quick decisions and moved to the correct and rewarded end of the chamber. However, when presented with two very near concentrations of the same odor which are difficult to tell apart, the flies take much longer to make a decision leading also to more mistakes. This increase in reaction time when faced with poor quality of sensory information indicates a decision-making process in their tiny brains (DasGupta et al., 2014). Furthermore, when fruit flies fly in a white and completely featureless arena, they express endogenously-created patterns of spontaneous behavior (Maye et al., 2007). This suggests a non-random endogenous process of behavioral choice, which might imply a precursor motif of “spontaneous” behavior (as opposed to reflexive behavior).

We will first address manipulations that affect an individual host. For the sake of clarity, we have classified these into three general categories: (1) those that affect the compass or navigation of the host that leads to a suicidal behavior. (2) Those which induce the so-called bodyguard behavior. (3) Those that affect the host motivation to move. Then, with some insect species being social and living in colony, we will address manipulations that affect the individual in a social context. Regarding the latter, we will highlight examples of manipulation where the individual, when infected, shows “antisocial” behavior.

## Suicidal Behavior

Some parasitic fungi and worms manipulate their host’s navigational system in most strange ways. Such manipulation ends with the suicide of the host. For example, an ant falling victim to parasitic fungus of the genus *Cordyceps* is manipulated to produce a behavior that facilitate dispersal of the fungus, thereby optimizing the parasite’s chances of reproduction (Hughes, 2015). To this end, *Cordyceps* fungi produce chemicals that alter the navigational sense of their ant hosts. It begins with the attachment of the spores of the fungus to the cuticle of the ant. The spores then germinate and break into the ant’s body by diffusing through the tracheae. Then, fungal filaments called mycelia grow by feeding on the host’s organs, avoiding, however, vital ones. The fungus then produces certain, yet unidentified, chemicals that cause the ant to climb to the top of a tree or plant and clamp its mandibles around a leaf or leaf stem to stay in place, a behavior that has never reported for uninfected ants. When the fungus is ready to produce spores, it eventually feeds on the ant’s brain and thus kills it. The fruiting bodies of the fungus then sprout out of the cuticle and release capsules filled with spores. The airborne capsules explode on their descent, spreading the spores over the surrounding area to infect other ants and thus start another cycle (Hughes et al., 2011).

Ants can also fall victim to another parasite with a strategy to facilitate the transmission from the intermediate host (the ant) to the final host (a grazing animal). The Lancet liver fluke (*Dicrocoelium dendriticum*) takes over the ant’s (*Formica fusca*) navigational skills to coerce it into climbing to the tip of a blade of grass (Hohorst and Graefe, 1961). In this position, the ant waits for its deadly fate: being eaten by a grazing animal. The cycle starts with the mature Lancet fluke housing in the liver of the grazing animal and producing eggs which are expelled in the digestive system of the grazer to end up in its feces. Snails get infected by feeding on such droppings. The fluke larvae settle in the snail to be in turn expelled in slime balls. Ants are fond of these slime balls and after a brief sojourn in the ant’s gut, the parasites infest the ant’s hemolymph and drift inside its body. Remarkably, only one of those parasites migrates alone to the ant’s head and settles next to one of the cerebral ganglia, the sub-esophageal ganglion. In this strategic location, it presumably releases some unknown chemicals to control the ant behavior. When evening approaches and the air cools, the infested ant leaves the colony and moves upward to the top of a blade of grass. Once there, it clamps its mandibles onto the top of the blade and stays, waiting to be devoured by some grazer. At the break of day, if the ant life was spared during the night, it returns to the ground and behaves normally. When evening comes again, the fluke takes control again and sends the ant back up the grass for another attempt until a grazing animal wanders by and eats the grass. And so begins a new cycle for the parasite.

Parasites are not necessarily phylogenetically distant from their host. For instance, the crypt gall wasp (*Bassettia pallida*) parasitizes oaks. It lays an egg in the stem and larva induces the development of a ‘crypt’ within growing stems. This ‘crypt’ serves as protection to the larva until it pupates and digs its way out of the stem. This parasitic wasp can be manipulated by another wasp: the parasitoid crypt-keeper wasp (*Euderus set*) (Weinersmith et al., 2017). When parasitized, adult gall wasps dig an emergence hole in the crypt wall as they do normally, however, instead of emerging through the hole, they plug the hole with their head and die. This benefits the parasite, instead of having to excavate an emergence hole of its own to avoid being trapped, it can use the host’s head capsule as an emergence. Dissections of head-plugged crypts reveal larval and pupal stages of the parasitoid residing partly within the crypt and partly within the host’s body.

**FIGURE 1:**
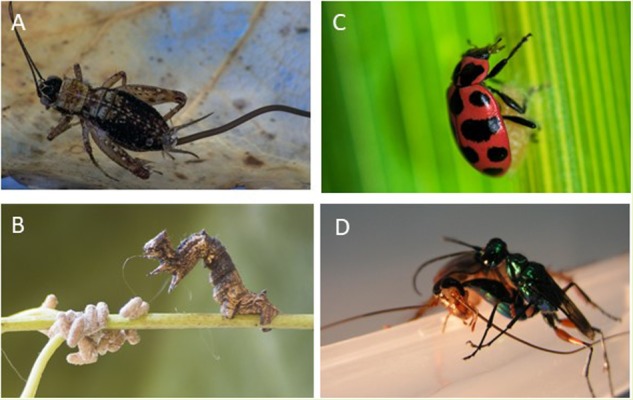
**(A)** A parasitic worm emerging from its drowning cricket host (Credit: Pascal Goetgheluck). **(B)** Ladybug guarding a wasp cocoon (Credit: Mathieu B. Morin). **(C)** Wasp manipulates caterpillar into serving as a bodyguard to it cocoons (Credit: Jose Lino-Neto). **(D)** Wasp injects venom into the brain of a cockroach to use it as a fresh food supply for its offspring (from the authors’ lab).

Crickets and other terrestrial insects can fall victim to hairworms, which develop inside their bodies and lead them to commit suicide in water, enabling the exit of the parasite into an aquatic environment favorable to its reproduction (**Figure 1A**). The mechanisms used by hairworms (*Paragordius tricuspidatus*) to increase the water-seeking behavior of their orthopteran hosts (*Nemobius sylvestris*) remain a poorly understood aspect of this manipulative process (Ponton et al., 2011). Results of two earlier proteomics studies suggest that phototaxis alterations (i.e., changes in the responses to light stimuli) could be a part of a wider strategy of hairworms for completion of their life cycles (Biron et al., 2005, 2006). Specifically, parasite-induced positive phototaxis could improve the encounter rate with water (Biron et al., 2006). This assumption was derived from two arguments. Firstly, in the native forest of southern France, water areas such as ponds and rivers are, at night, luminous openings contrasting with the dense surrounding forest. Thus, light could then be a sensory cue that leads infected arthropods to an aquatic environment (Henze and Labhart, 2007). Secondly, besides this ecological reasoning, proteomics data reveal a differential expression of protein families that may be functional components of the visual cycle in the central nervous system of crickets harboring hairworms (Biron et al., 2006).

## Offspring Care

Although solitary insects are not known to provide care and safety to their offspring, one of the most fascinating behavioral manipulations of parasites is to coerce a host to care for the parasite’s offspring. This manipulation is known in insect parasitoids and consists in coercing the host in providing protection to the parasite’s offspring from predators (the so-called “bodyguard manipulation”). Protection of this form has been reported for various caterpillar-wasp associations. First, the wasp (A member of the *Glyptapanteles* species) stings and injects her eggs into the caterpillar (*Thyrinteina leucocerae*) (Grosman et al., 2008). The caterpillar quickly recovers from the attack and resumes feeding. The wasp larvae mature by feeding on the host, and after 2 weeks, up to 80 fully grown larvae emerge from the host prior to pupation. One or two larvae remain within the caterpillar while their siblings perforate the caterpillar body and begin to pupate. After emergence of the larval wasps to pupate, the remaining larvae take control of the caterpillar behavior by an unknown mechanism, causing the host to snap its upper body back and forth violently, deterring predators and protecting their pupating siblings (**Figure 1B**). Un-parasitized caterpillars do not show this behavior. This bodyguard behavior results in a reduction in mortality of the parasitic wasp offspring. Interestingly, this aggressive behavior of the caterpillar toward intruders must be a component of the host’s behavioral repertoire that is usurped by the parasitoid to fulfill another purpose beneficial to the wasp.

Another species of wasp manipulates its host even after leaving the host’s body. In the exquisite manipulation, the wasp (*Dinocampus coccinellae*) inserts one egg only into a ladybug (*Coleomegilla maculata*) and after emergence of the larva, the ladybug guards the cocoon (Maure et al., 2013). Initially, the single wasp larva develops inside the body of its host, but after about 20 days, it emerges from the ladybug’s body and spins a cocoon between its legs. Once the wasp larva has emerged, the ladybug remains alive on top of the cocoon (**Figure 1C**), twitching its body to keep the single wasp pupa safe from potential predators such as lacewings (Dheilly et al., 2015). The survival rate of cocoons protected by living ladybugs from a lacewing predator (another insect) is roughly 65%. If cocoons are left unprotected or attached to dead ladybugs, none or at best 15% survive. Thus, the ladybug, as a bodyguard of the wasp offspring is similar in function to that of the preceding example. Given that the wasp pupa is outside of the ladybug body, and no siblings remain inside the ladybug body, how does this manipulation occur? It appears the wasp injects together with an egg, a virus. The larval-stage parasite contains the virus, and just before the larva exits the host to pupate (and benefits from the bodyguard behavior), it experiences a massive increase in viral replication which are transmitted to the ladybug. The virus replication in the host’s nervous tissue induces a severe neuropathy and antiviral immune response that correlates with the symptoms characterizing the motor twitches that serve to protect the pupa (Dheilly et al., 2015). Hence, the virus is apparently responsible for the behavioral change because of its invasion of the ladybug’s brain and the virus clearance correlates with behavioral recovery of the host.

On the surface, the interactions between the caterpillar (*Narathura japonica*) and the ants (*Pristomyrmex punctatus*) looks like an evolved mutualism (an association between two organisms of different species that beneficial to both organisms). But with a closer look, the caterpillar, which is tended by ants, provides the ants with a secreted substance (sugar-rich secretions) which makes the attendant ants more aggressive. When more aggressive, the ants are less likely to move away from the caterpillar, thereby reducing the chances that the caterpillar would be targeted by predators (Hojo et al., 2015). Although the caterpillar does not invade the ant’s body, the researchers found elevated levels of Dopamine in the ant’s nervous system.

## Spontaneity

The neuronal underpinnings responsible for behavioral spontaneity in insects remain elusive. In our laboratory, we are exploring a unique and naturally-occurring phenomenon in which one insect uses neurotoxins to apparently “hijack” the decision-making ability of another. This phenomenon, a result of millions of years of co-evolution between a cockroach and its wasp parasitoid, offers a unique opportunity to study the roots and mechanisms of spontaneous behavior in non-human organisms. So far, our investigations point to one possible neuronal substrate involved in the regulation of spontaneous behavior in insects.

The cockroach central nervous system comprises two cerebral ganglia in the head, the supraesophageal ganglion (‘brain’) and the subesophageal ganglion (SEG). The cerebral ganglia have been implicated in controlling expression of locomotor patterns that are generated in the thoracic ganglia (Kien and Altman, 1992; Schaefer and Ritzmann, 2001). The thoracic ganglia house networks of inter- and motoneurons, which, among other functions, generate the motor patterns for flight and walking. In the brain, numerous investigations suggest that a central structure called the central complex (CX), which is involved in sensory integration and pre-motor processing, is also involved in ongoing regulation of locomotion. For instance, in cockroaches, some CX units show increased firing rates preceding initiation of locomotion and stimulation of the CX promotes walking, indicating that the CX is predominantly permissive for walking (Bender et al., 2010). The Jewel Wasp (*Ampulex compressa*) stings cockroaches (*Periplaneta americana*) (**Figure 1D**) and injects venom into the SEG and in and around the CX in the brain (Haspel et al., 2003). The venom induces a long-term hypokinetic state characterized by the inability of the stung cockroach to initiate walking. Other behaviors such as righting, flying, or grooming are not affected. Although stung cockroaches seldom express spontaneous or evoked walking under natural conditions, immersing them in water is stressful enough to induce spontaneous coordinated walking similar to that observed in un-stung cockroaches. However, stung cockroaches maintain swimming for much shorter durations than un-stung cockroaches, as if they ‘despair’ faster (Gal and Libersat, 2008). This and other examples suggest that the venom selectively attenuates the ongoing ‘drive’ of cockroaches to produce walking-related behaviors, rather than their mechanical ability to do so. Our recent data indicate that behavioral manipulation of cockroaches by the jewel wasp is achieved by venom-induced inhibition of neuronal activity in the CX and SEG. Our results show that focal injection of procaine or venom into the CX is sufficient to induce a decrease in spontaneous walking indicating that the CX is necessary for the initiation of spontaneous walking. Furthermore, venom injection to either the SEG or the CX of the brain is, by itself, sufficient to decrease walking initiation (Gal and Libersat, 2010; Kaiser and Libersat, 2015). Hence, our investigation of the neuronal basis of such parasite-induced alterations of host behavior suggests that the parasite has evolved ways to tap on the host’s brain circuitry responsible for behavioral spontaneity.

## Sociality

The organization of insect sociality implies cooperative care of offspring and a division of labor into different castes each with a specific task for the benefit of the society (Michener, 1969). This complex organization can be penetrated by specialist “social parasites” (Barbero et al., 2009). One such parasite is the caterpillar (*Maculinea rebeli*) which mimics the ants (*Myrmica schencki*) surface chemistry and the sounds they use to communicate, allowing it to penetrate the ant colonies undetected and enjoy the treats of their queen larvae (Akino et al., 1999; Thomas and Settele, 2004). Ironically, those social parasites are the victims of a parasitoid wasp (*Ichneumon eumerus*) which deposits its eggs into the caterpillar. The wasp’s offsprings emerge later as adults from the caterpillar cocoon. The wasp seeks the caterpillar host by first detecting the ant colonies. The body surface chemicals expressed by the wasp induce aggression in ants, leading to in-fighting between the ants. This distraction permits the wasp to penetrate the nest and attack the caterpillar host.

In fire ant parasitic flies (*Pseudacteon tricuspis*), the female will strike an ant and inject an egg into the ant’s (*Solenopsis invicta*) body. After the larva hatches, it moves into the ant’s head and feeds mostly on hemolymph (the equivalent of blood in insect) until just prior to pupation. The larva then consumes the contents of the ant’s head, upon which the head usually falls free of the body. The adult fly will emerge from the ant’s head 2–6 weeks after pupation. Unlike un-parasitized ants which die inside the nest, those parasitized by the fly larvae leave the safety of the nest shortly before their decapitation. Yet, when parasitized ants leave their nest prior to decapitation, their behavior is indistinguishable from un-parasitized ants. The host’s brain is evidently still intact when the ants leave the colony as it is last consumed by the parasitoid (Henne and Johnson, 2007).

From ants to honeybees; Microsporidia (*Nosema ceranae*), a unicellular parasite, infection in honey bees (*Apis mellifera*) affects a range of individual and social behaviors in young adult bees (Lecocq et al., 2016). In social bees, age polyethism refers to the functional specialization of different members of a colony based on age. Infection of bees by the parasite significantly accelerates age polyethism causing them to exhibit behaviors typical of older bees. Infected bees also have significantly increased walking rates and higher rates of trophallaxis (food exchange) (Lecocq et al., 2016).

Switching from social bees to social wasps, a fly-like larva (*Xenos vesparum*) waits for a wasp (*Polistes dominula*) to land nearby and strikes, penetrating the wasp cuticle to dwell into its abdomen and feeds on its blood (Beani, 2006). Paper wasps are eusocial animals, the highest organization of sociality in animals. When infected with the fly parasite, the normally social wasp starts withdrawing from its colony showing some erratic behavior for no apparent reason other than the presence of the parasite inside it body, messing up with its brain (Hughes et al., 2004). Eusocial colonies include two or more overlapping generations, show cooperative brood care and are divided into reproductive and non-reproductive castes. Individuals of at least one caste usually lose the ability to perform at least one behavior characteristic of individuals in another caste (Michener, 1969). Paper wasp colonies are founded in the spring by one or several females gynes (non-working pre-overwintering queens), who build the nest and rear a first generation of female workers. The founding female will become the primary reproductive colony queen, while the workers perform tasks such as nest building and brood care. Later in the colony cycle, larvae are reared by workers and emerge as males or female gynes. Those gynes leave the colony in the fall to form aggregations outside the colony with other gynes, where they spend the winter until they scatter to find new colonies in the spring. Female wasps infested by the fly-like larva undergo dramatic behavioral changes. Although those females should be workers they behave as typical gynes: they show nest desertion and formation of pre-overwintering aggregations. This behavior is beneficial for the mating and distribution of the parasite (Hughes et al., 2004). In early summer, the infected wasp just leaves its colony behind on a journey to a meeting place with other infected wasps. Male and female parasites can then mate. Whereas wasps infected by male flies die, those infected by females remain alive and under the control of their parasites. They begin to act like wasp zombie queens feeding and growing until they go back to their or other colonies loaded with fly larvae to infect their sister wasps. RNA-sequencing data used to characterize patterns of brain gene expression in infected and non-infected females shows that infected females show gyne brain expression patterns. These data suggest that the parasitoid affects its host by exploiting phenotypic plasticity related to social caste, thus shifting naturally occurring social behavior in a way that is beneficial to the parasitoid (Geffre et al., 2017).

## Conclusion

For comparison, the best-studied example of parasitic manipulation of cognitive function in mammals is the case of Toxoplasmosis, an illness caused by the protozoan parasite *Toxoplasma gondii*. It infects rodents such as mice and rats (the intermediate host) to complete its life cycle in a cat (the final host). The parasite infects the brain forming cysts that produce an enzyme called tyrosine hydroxylase, the limiting enzyme to make dopamine. The most conspicuous behavioral modification in the rat is a switch from avoidance to attraction to cat urine (Berdoy et al., 2000). In doing so, the parasite facilitates its own transmission from the intermediate host to the final host. Such a specific behavioral changes suggests that the parasite finely modify the brain neurochemistry of its intermediate host to facilitate predation, leaving other behavioral traits untouched. This has led to the hypothesis that the host brain is overflown with excess dopamine produced by the parasite, hence, making dopamine the primary suspect in this manipulation. Recently, the parasite genes that encode tyrosine hydroxylase have been identified. By generating a tyrosine hydroxylase mutant parasitic strain of toxoplasma, it was possible to test directly the involvement of dopamine in the manipulation process (Afonso et al., 2017). The authors reported that both mice infected with wild type or mutant (enzyme deficient) strains showed both changes in exploration/risk behavior.

Although humans are dead-end host for the parasite, humans can be infected and some scientists have suggested that *T. gondii* infection can alter human behavior. Because the parasite infects the brain, it is suspected of making people more reckless, even being liable for certain cases of schizophrenia (Fuglewicz et al., 2017). However, such a hypothesis is still highly controversial and will require more investigations. Today, modern humans are not suitable intermediate hosts because big cats no longer prey upon them. Hence, behavioral modifications in humans could represent a residual manipulation that evolved in appropriate intermediate hosts. An alternative hypothesis, however, states that these changes result from parasite manipulative abilities that evolved when human ancestors were still under significant feline predation. In order to understand the origin of such behavioral change in humans, a recent study tested chimpanzees, which are still preyed upon in their natural environment by leopards. The behavioral test centered on olfactory cues showed that, whereas uninfected individuals avoided leopard urine, parasitized individuals lost this aversion (Poirotte et al., 2016). In the frame of the human evolution, hominids have long coexisted with large carnivores and were considered as good as a meal as our distant and extinct cousins. Hence, when big cats were chasing our ancestors, *T. gondii* manipulative skills could have evolved because early hominids were suitable intermediate hosts.

Beyond the awe with which we observe the amazing parasitic manipulations described in this review, there is a need to investigate the proximate mechanisms of such behavioral manipulations. Although our understanding of the neural mechanisms of parasitic manipulation is still in its infancy, there have been some major progresses mostly due to advances in molecular biology, biochemistry and biological engineering. Even with tiny quantities of the parasite’s secretome (secretions produced by the parasite that may be involved in the host nervous system manipulation), we can use metabolomic, proteomic, and transcriptomic approaches to characterize the library of the secretome components. However, deciphering the composition of the parasite secretome is only the first necessary step. The next and more challenging step is to determine a causal relationship between individual secretome components and their contribution to the observed behavioral manipulation of the host. One promising avenue to address this challenge relies on the recent availability of gene editing tools such as RNA interference (a method of silencing gene product for editing the secretome content) and CRISPR Cas-9 (a method for editing parts of the genome in the parasite). By combining these tools, we are getting closer to unravel the molecular mechanisms of these extraordinary behavioral manipulations.

## Author Contributions

All authors listed have made a substantial, direct and intellectual contribution to the work, and approved it for publication.

## Conflict of Interest Statement

The authors declare that the research was conducted in the absence of any commercial or financial relationships that could be construed as a potential conflict of interest.
